# Development and Application of Magnetically Controlled Capsule Endoscopy in Detecting Gastric Lesions

**DOI:** 10.1155/2021/2716559

**Published:** 2021-12-30

**Authors:** Yaoping Zhang, Yanning Zhang, Xiaojun Huang

**Affiliations:** ^1^Department of Gastroenterology, Lanzhou University Second Hospital, Lanzhou, Gansu Province, China; ^2^Gansu Provincial Digestive Endoscopy Engineering Research Center, Lanzhou, Gansu Province, China

## Abstract

In the past 20 years, several magnetically controlled capsule endoscopes (MCCE) have been developed for the evaluation of gastric lesions, including NaviCam (ANKON), MiroCam-Navi (Intromedic), Endocapsule MGCE (Olympus and Siemens), SMCE (JIFU), and FAMCE (Jinshan). Although limited to observing esophageal and duodenal lesions and lacking the ability of biopsy, MCCE has the advantages of comfort, safety, no anesthesia, no risk of cross-infection, and high acceptability. Several high-quality RCTs showed that the diagnostic accuracy of MCCE is comparable to the traditional gastroscopy. Due to the nonnecessity of anesthesia, MCCE may be more suitable for the elderly with obvious comorbidities as well as children. With more evidences accumulated and more innovative technologies developed, MCCE is expected to be an important tool for screening of early gastric cancer or the diagnosis of gastric diseases.

## 1. Introduction

Gastric diseases, including gastritis, gastric ulcer, and gastric cancer, are common in the general population, which seriously threaten human health and cause a huge economic burden. The newly released Global Cancer report shows that there are an estimated 1.033 million new cases of gastric cancer and 783,000 deaths in 2018, ranking fifth in the incidence of malignant tumors and third in the causes of death [1]. According to the 2015 Chinese Cancer Statistics, it is estimated that there are 679,000 new cases of gastric cancer and 498,000 deaths in China each year, accounting for about 42.6% and 45.0% of the world, and the incidence and mortality of gastric cancer in China are the second highest among malignant tumors [2].

Correa and Piazuelo [3] proposed that most of the occurrence of gastric cancer involves a series of pathological changes, including atrophic gastritis, intestinal metaplasia, dysplasia, and adenocarcinoma, and the risk of gastric cancer gradually increases in this series of cascade reactions [4]. Most patients with gastric cancer and precancerous lesions have no specific symptoms. Due to the lack of effective early screening methods, the early diagnosis and treatment rate of gastric cancer in China is less than 10%, and the 5-year survival rate is low (31.3%), which is much lower than that of Japan and South Korea [5–7]. Therefore, early diagnosis and treatment of gastric cancer and precancerous lesions are essential to reduce the mortality associated with gastric cancer and prolong the survival time of patients with gastric cancer.

At present, traditional gastroscopy is considered to be the best way to detect gastric diseases. However, due to the uncomfortable and inconvenient examination process of unsedated gastroscopy, patients may refuse necessary endoscopy [8, 9]. Although painless gastroscopy is highly accepted by patients, it increases costs and risks [10]. In addition, due to the limitation of endoscopic equipment and experienced physicians, its application in the screening of gastric diseases in the common population is limited. Therefore, a simpler and less invasive inspection method is needed as an alternative to traditional gastroscopy to increase the rate of early diagnosis and treatment of gastric diseases in the population.

Since its birth in 2000 [11], capsule endoscopy has been widely used in the diagnosis and treatment of small intestinal diseases, such as small intestinal bleeding, Crohn's disease, small intestinal malignant tumors, and small intestinal mucosal lesions associated with nonsteroidal anti-inflammatory drugs [12]. It is now a routine technique carried out by digestive endoscopists. In recent years, with the continuous development of capsule endoscopy technology, esophageal capsule endoscopy, colon capsule endoscopy, and magnetically controlled capsule endoscopy (MCCE) dedicated to the examination of gastric diseases have also been developed one after another [13–15]. This review reviews the current research on the application of MCCE in the examination of gastric disease. The prospects for future development of MCCE are also discussed.

## 2. The Type of MCCE

The concept of magnetically controlled capsule endoscopy was first proposed by Carpi et al. in 2006 [16]. It is mainly used for the observation of the gastric cavity. At present, there are three main types of MCCE in clinical application: handle-type, MRI-type, and robotic-type (Table 1).

### 2.1. Handheld-Type MCCE

In 2010, Swain et al. [17] reported for the first time that a handheld magnetically controlled capsule endoscopy was applied to a volunteer. Under the monitoring of a traditional gastroscopy, they demonstrated the movement of the MCCE in the human esophagus and stomach cavity. The MCCE is based on the transformation of the colon capsule endoscope (Given Imaging Ltd., Yoqne'am, Israel). A neodymium-iron-boron magnet is installed at one end of the capsule, which can operate normally and transmit images under the control of an external magnetic field. The handheld external magnet consists of two rectangular plate-shaped magnets and a handle, with a size of 10 × 10 × 3 cm, which is used to control the capsule endoscopy in the esophagus and stomach. The MCCE has the advantages of relatively simple and low manufacturing cost and seems to be safe to be applied to the human body, and the subjects do not have any discomfort. In the same year, Keller et al. [18] conducted a feasibility study to further evaluate the safety and effectiveness of handheld MCCE in the esophagus of healthy people without the aid of a supervising gastroscopist. The results showed that it was safe and feasible to use the handheld MCCE to examine the esophagus of healthy volunteers, but it may require greater magnetic force. The handheld MCCE system modified by Keller et al. [19] consists of four main subsystems: an ingestible capsule endoscope, a data recorder, a RAPID Real-Time Viewer (RTV), and a computer workstation with RAPID software (all from Given Imaging Ltd., Yoqne'am, Israel). The handheld external magnet weighs 530 g and can generate a maximum magnetic force of 272 g/cm^2^. They performed operations on 10 healthy volunteers to evaluate the effectiveness and safety of their examination in the human stomach. The results showed that 7 cases had good mucosal visibility (75%~90%), and the remaining 3 cases had moderate degree of visibility (50%~60%). No adverse events occurred. It is safe and feasible to use a handheld magnet to remotely control the capsule endoscopy in the stomach of healthy volunteers. The system has clinical application value and is worthy of further development.

Based on the modification of the MiroCam small intestine capsule endoscopy, Intromedic (Seoul, Korea) developed another handheld-type MCCE system called the MiroCam-Navi. Hale et al. compared it with traditional gastroscopy on ex vivo porcine stomachs, and the two methods achieved comparable results [20]. Like the standard MiroCam small intestine capsule endoscope, the signals and images of the MiroCam-Navi are sent to the receiver through a novel transmission technology called electric field propagation (rather than radio frequency), which uses the human body as a communication medium [21]. In order to evaluate the maneuverability of the MiroCam-Navi in the upper gastrointestinal tract of humans, Rahman et al. [22] recruited 26 volunteers for clinical trials. The study showed that the visualization of the main anatomical landmarks of the upper gastrointestinal tract reached 88% to 100% (esophagogastric junction 92%, cardia 88%, fundus 96%, body 100%, incisura 96%, antrum 96%, and pylorus 100%). However, it is worth noting that the *Z*-line was only visualized in 46% of cases; it is difficult to control the speed of the capsule entering the stomach. Moreover, the magnetic field of the handheld magnet is weak, so the ability to observe gastric mucosa closely and accurately control the capsule needs to be further improved. In order to achieve complete visualization of the upper gastrointestinal mucosa, Rahman et al. [23] used a multiplanar reconstruction CT modelling to determine the best position of the magnetic capsule endoscope in the upper gastrointestinal tract and determine the best position of a handheld magnet to help it pass through the pylorus. In terms of diagnostic accuracy, studies have shown that MiroCam-Navi can be used in the examination of Barrett's esophagus, esophageal varices, acute upper gastrointestinal bleeding, and recurrent or refractory iron deficiency anemia, showing good diagnostic ability and high tolerance [24–26].

### 2.2. MRI-Type MCCE

In 2010, Rey et al. [27] reported a magnetically navigated video capsule endoscope (MGCE), developed by Olympus Medical Systems Corporation and Siemens Healthcare, which was a capsule endoscopy system based on the nuclear magnetic resonance system. The system includes an Olympus capsule endoscope and a Siemens magnetic guidance device for interactively moving the capsule in the gastric cavity. The capsule endoscope is 31 mm long and 11 mm in diameter. It is equipped with two image sensors that use a charge-coupled device (CCD). It captures and records images from both the forward and backward directions of the capsule movement with a frame rate of 4 frames per second. Like the small intestine capsule, the patient is equipped with multiple antennas to record images of the MGCE capsule. Standard imaging is performed in real time. The images from the two sensors are displayed on one display of the dual display panel at the same time. The magnetic guidance equipment is similar to MRI in appearance, covering an area of 1 m × 2 m, but the magnetic field to mobilize the capsule endoscope is much smaller (the magnetic field of traditional MRI scanning is 150-500 times of this magnetic field), and the maximum magnetic field is 100 mT, so its potential side effects are lower. The capsule can be magnetically steered with five mechanical degrees of freedom (translation in three dimensions, rotation, and tilting). This study was the first systematic trial conducted in 29 volunteers and 24 patients. The technical success rate was 98%. The visualization rates of the gastric pylorus, antrum, body, fundus, and cardia were 96%, 98%, 96%, 73%, and 75%, respectively. The potential of MRI-type MCCE has been clearly proven in this experiment, and its feasibility, visualization of the gastric lesion, and safety are high. In 2012, Rey et al. [28] firstly conducted a blinded control study of conventional gastroscopy and MGCE on patients with upper gastrointestinal symptoms. 71patients with indications for gastroscopy participated in the study, of which 61 patients completed the MGCE examination. The results showed that the visualization rates of the gastric pylorus, antrum, body, fundus, and cardia were 88.5%, 86.9%, 93.4%, 85.2%, and 88.5%, respectively. In addition to the 63 lesions that were detected by both gastroscope and MGCE, the gastroscope found another 14 lesions that were not detected by the MGCE, and the MGCE detected 31 lesions that were missed by the gastroscope. The diagnosis results of the two methods are similar.

In 2015, Denzer et al. [29] carried out a prospective blind study to systematically evaluate the accuracy of MGCE in the diagnosis of major gastric lesions (defined as lesions that require conventional gastroscopy for biopsy or resection) in patients with upper gastrointestinal symptoms. Examination of the esophagus and duodenum was not included in this comparative study. Among 189 symptomatic patients, a total of 23 major lesions were found in 21 patients. The accuracy of MGCE was 90.5% [95% confidence interval (CI), 85.4%-94.3%] with a specificity of 94.1% (95% CI, 89.3%-97.1%) and a sensitivity of 61.9% (95% CI, 38%-82%). Among the remaining 168 patients, 94% had minor and mostly multiple lesions, and the diagnostic accuracy of MGCE was 88.1% (95% CI, 82.2%-92.6%). All patients preferred MGCE.

### 2.3. Robotic-Type MCCE

In 2012, Liao et al. [30] first reported a robotic-type magnetically controlled capsule endoscopy (MCE) system, developed by Ankon Technologies Co. Ltd. The system includes a capsule endoscope, a guidance magnet robot, a data recorder, and a computer workstation with software for real-time view and control. In January 2013, it was certified by the China Food and Drug Administration (CFDA) and became the first magnetically controlled capsule gastroscopy system to be marketed in the world. The size of the capsule is 28 × 12 mm, and the image is captured at a rate of 2 frames per second and transmitted to the data recorder through a set of sensors placed on the patient's skin. The guidance magnet robot is of C-arm type which can generate a magnetic field up to 200 mT, much smaller than the magnetic field produced by standard 1.5 T magnetic resonance imaging. Through the precise robotic-type external magnetic field control system, the capsule can be easily controlled with five degrees of freedom—two rotational and three translational. The highlight lies in the millimeter-level (2 mm) movement in the three-dimensional linear direction and the free and small angle (3°) rotation of the capsule endoscope, so as to facilitate accurate movement of the capsule endoscope to any part of the three-dimensional cavity of the stomach. Liao et al. [30] reported a preliminary study in healthy volunteers to evaluate the feasibility and safety of the MCE system in human stomach examinations. The results showed that 79.4% of patients showed a gastric mucosal visualization rate at 75%, and the visualization rates of gastric cardia, fundus, body, angle, antrum, and pylorus were 82.4%, 85.3%, 100.0%, 100.0%, 100.0%, and 100.0%, respectively. The patients had a high degree of acceptance in the preparation before and during the examination, and there were no adverse events. Zou et al. [31] further compared the diagnostic accuracy of the MCE system and standard gastroscopy in the diagnosis of gastric diseases. 68 patients received standard gastroscopy 4-24 hours after MCE examination. The results showed that the positive percentage agreement between MCE and gastroscopy was 96.0%, the negative percentage agreement was 77.8%, and the overall agreement was 91.2% with a kappa value of 0.765 (*P* < 0.001). The results indicate that MCE shows similar diagnostic accuracy to standard gastroscopy; it may be a promising alternative to gastroscopy for noninvasive screening of gastric diseases. In 2016, a multicenter clinical trial involving seven hospitals in China showed that with traditional gastroscopy as the gold standard, the sensitivity, specificity, positive predictive value, and negative predictive value of MCE for detecting focal gastric lesions were 90.4%, 94.7%, 87.9%, and 95.9%, respectively, and its accuracy was comparable to that of traditional gastroscopy. Compared with traditional gastroscopy, almost all patients prefer MCE. It can be seen that MCE can be used for gastric disease screening without sedation [32]. In 2017 and 2018, several medical associations in China jointly formulated “The China Expert Consensus of Clinical Practice for Magnetically Controlled Capsule Gastroscopy (2017, Shanghai)” [33] and “Technological Specification for Medical Quality Control of Magnetically-Controlled Capsule Gastroscopy System” [34]. The indications and contraindications of magnetically controlled capsule endoscopy were defined, and the operation procedure was standardized.

In the diagnosis of gastric tumors, magnetically controlled capsule endoscopy also has a good performance. In 2018, Qian et al. [35] conducted a preliminary study to explore the feasibility of magnetically controlled capsule gastroscopy (MCCG) to detect superficial gastric tumor lesions. The study included 10 patients who had been diagnosed with superficial gastric tumors and planned to undergo ESD surgery and received MCCG examination the day before surgery. The results showed that the sensitivity of each patient and lesion detected by MCCG for superficial gastric tumor was 100% and 91.7%, respectively. The endoscopic findings of these lesions observed by MCCG and traditional gastroscopy are in good agreement. In addition, a number of studies have evaluated the value of MCCG in gastric cancer screening in asymptomatic people. The results show that MCCG is safe, noninvasive, effective, and economical, and it is feasible to be used in large populations [36–38].

MCCG also has certain application value in elderly patients and minor patients. In 2019, a study by Zhang et al. [39] explored the effectiveness and safety of MCCG in elderly patients. The preliminary results show that MCCG is of considerable benefit to the elderly and is generally safe. Another study by Gao et al. [40] shows that MCCG can monitor aspirin-related gastric mucosal lesions in elderly patients and guide treatment decisions. In 2018, a clinical study in Ruijin Hospital Affiliated to Shanghai Jiaotong University recruited 84 minor patients hospitalized in the pediatric department who underwent MCCG examinations, with an average age of 12 ± 3.2 years. The results showed that the diagnosis rate of MCCG for gastric diseases was 13.1%, the sensitivity was 13.1%, and no adverse events occurred [41]. To explore the feasibility and safety of the application of MCCG in children, a retrospective study in Shanghai Children's Hospital included 129 children with gastrointestinal symptoms who needed gastroscopy but were unwilling or unable to tolerate routine gastroscopy (including painless gastroscopy), aged 6 to 14 years old, with an average of 9.8 ± 1.9 years old. The results show that MCCG is feasible and safe in children over 6 years old, and more studies are needed to further study the efficacy of MCCG in children [42]. Xie et al. [43] performed MCCG examinations on 48 patients with abdominal pain aged 6-18 years to evaluate the diagnostic effects. The results showed that the visualization rates of the cardia, fundus, body, angle, antrum, and pyloric mucosa were 84.8%, 83.8%, 88.5%, 87.7%, 95.2%, and 99.6%, respectively. Among them, 19 gastrointestinal lesions were found in 18 patients: 1 esophageal lesion, 3 gastric lesions, and 15 small intestinal lesions. No adverse events occurred during the follow-up period.

In 2020, Jiang et al. [44] reported the second-generation magnetically controlled capsule gastroscope (MCCG-2) with higher image resolution (720 × 720) and adaptive frame rate (8 fps) and conducted a randomized controlled clinical trial with the first-generation magnetically controlled capsule gastroscopy (MCCG-1) to evaluate its application value in the upper gastrointestinal examination. The results show that MCCG-2 shows better performance in mucosal visualization, examination duration, and maneuverability, so it can better diagnose upper gastrointestinal diseases. In addition, Cheng et al. [45] reported a standing-type magnetically controlled capsule endoscopy system (SMCE), developed by Fuzi Technologies Co. Ltd. (Shenzhen, China), which consists of four parts: an ingestible capsule endoscope, a guidance magnet robot, a data recorder, and a computer workstation with software for real-time viewing and control. The size of the capsule is 27 mm × 12 mm, the depth of field is 0-50 mm, the viewing angle is 136°, the image resolution is 480 × 480, and the frame rate is 4 frames per second. Under the guidance of the magnetic navigation robot, the capsule can perform six basic actions, including forward, backward, ascending, descending, rotating, and flipping. The magnetic navigation robot integrates a data recorder with an antenna. The image signal from the capsule is transmitted directly to the antenna of the data recorder, thus avoiding the commonly used wired skin contact sensor. The images are displayed and stored in real time on the workstation monitor at the same time. A pilot study evaluated the feasibility and safety of the SMCE system in 31 healthy volunteers. The results showed that all subjects (100%) could well visualize the anatomical landmarks of the stomach, including cardia, fundus, body, angle, antrum, and pylorus, and the mucosal visualization rate was more than 75% [45]. Gastric MCE examination in the standing position is feasible and safe and has good maneuverability. Lai et al. [46] conducted a prospective multicenter blinded study, using gastroscopy as the standard to evaluate the value of SMCE in detecting gastric lesions. The study included 161 patients with abdominal symptoms from three centers who underwent SMCE examination before painless gastroscopy. The results showed that the positive compliance rate between SMCE and gastroscopy was 92.0% (95% CI: 80.77%~97.78%). The negative compliance rate was 95.5%, and the overall compliance rate was 94.41%. No capsule retention or adverse events occurred.

However, due to the large size, high cost, and operator reliance of the robotic MCCG, its wider adoption at different health-care service levels and settings is limited [47]. In 2021, Xiao et al. [48] reported a fully automated magnetically controlled capsule endoscopy (FAMCE) system provided by Jinshan Technologies (Chongqing, China). The FAMCE system consists of a magnetically controlled capsule, an image recorder, a robotic arm capsule controller, and a workstation with software. The magnetically controlled capsule was 512 × 512 in resolution, with a field of view of 160°, a depth view of 0-50 mm, an adaptive image capture frequency of 2-8 fps, and a battery life of 9 h, which enables gastroscopy and a complete small bowel examination. The robotic arm capsule controller consisted of two magnetic fields and can automatically guide a magnetic controlled capsule to examine entire gastric cavity without requiring an operator to control the capsule manually. A prospective comparative study evaluated the efficacy and safety of FAMCE in the clinical application of gastroscopy and small bowel examination [48]. The results showed that the rate of complete detection of gastric anatomy landmarks (cardia, fundus, body, angulus, antrum, and pylorus) by FAMCE was 100% (95% CI 99.3-100.0). The concordance between FAMCE and conventional gastroscopy was 99.61% (99.45-99.78). And no adverse events occurred.

## 3. Conclusion

In summary, magnetically controlled capsule endoscopy has certain diagnostic capabilities and minimal invasiveness and can be applied to the examination of gastric diseases, which will be beneficial to the general screening of gastric cancer in large-scale populations. A number of studies have proven that the accuracy of the magnetically controlled capsule endoscopy for the diagnosis of gastric diseases is highly consistent with that of traditional gastroscopy. It has the advantages of comfort, safety, no need for anesthesia, and no risk of cross-infection and is highly accepted by the population. Especially during the COVID-19 epidemic, MCCE could reduce the risk of infection transmission compared with conventional gastroscopy. However, due to the lack of biopsy and treatment functions and the inability to inject gas and water, the magnetically controlled capsule endoscopy cannot replace the traditional gastroscopy at present. In the future, with the continuous updates and breakthroughs of science and technology, it is believed that magnetically controlled capsule endoscopy with more perfect functions will be developed, which will make a significant contribution to the screening of upper gastrointestinal diseases.

## Figures and Tables

**Table 1:** Comparison of different-type magnetically controlled capsule endoscopy.

Manufacturer	Given Imaging	Intromedic MiroCam-Navi	Olympus & Siemens	Ankon MCCG-1	Ankon MCCG-2	JIFU SMCE	Jinshan FAMCE
Year	2010	2015	2010	2012	2020	2019	2021
Control method	Handheld	Handheld	MRI guidance	Robotic control	Robotic control	Robotic control	Robotic control
Maximum magnetic field	272 g/cm^2^	341 mT	100 mT	200 mT	200 mT	200 + 50 mT	—
Size	11 × 31 mm	11 × 24 mm	11 × 31 mm	12 × 28 mm	11.6 × 26.8 mm	12 × 27 mm	—
Weight	7 g	4.2 g	—	5 g	5 g	2.7 g	—
Camera	1	1	2	1	1	1	1
Resolution	256 × 256	320 × 320	512 × 512	480 × 480	720 × 720	480 × 480	512 × 512
Frames	4 fps	3 fps	4 fps	2 fps	Adaptive 8 fps	4 fps	Adaptive 2-8 fps
Field of view	156°	175°	>145°	140°	150°	136°	160°
Depth of view	0-30 mm	0-30 mm	>20 mm	0-30 mm	0-30 mm	0-50 mm	0-50 mm
Battery life	10 h	8 h	—	>8 h	>12 h	30-40 min	9 h

## Abbreviations

MCCE: Magnetically controlled capsule endoscopy
RCTs: Randomized controlled trials
RTV: RAPID Real-Time Viewer
MGCE: Magnetically navigated video capsule endoscopy
CCD: Charge-coupled device
MCE: Magnetically controlled capsule endoscopy
CFDA: China Food and Drug Administration
MCCG: Magnetically controlled capsule gastroscopy
MCCG-1: First-generation magnetically controlled capsule gastroscopy
MCCG-2: Second-generation magnetically controlled capsule gastroscopy
SMCE: Standing-type magnetically controlled capsule endoscopy system
FAMCE: Fully automated magnetically controlled capsule endoscopy.
